# Serum levels of reactive oxygen metabolites at 12 weeks during tocilizumab therapy are predictive of 52 weeks-disease activity score-remission in patients with rheumatoid arthritis

**DOI:** 10.1186/s41927-019-0096-1

**Published:** 2019-12-16

**Authors:** Arata Nakajima, Keiichiro Terayama, Masato Sonobe, Yasuchika Aoki, Hiroshi Takahashi, Yorikazu Akatsu, Junya Saito, Shinji Taniguchi, Manabu Yamada, Ayako Kubota, Koichi Nakagawa

**Affiliations:** 1 Department of Orthopaedics, Toho University Sakura Medical Center, 564-1 Shimoshizu, Sakura, Chiba, 285-8741 Japan; 2 Department of Rheumatology, Toho University Sakura Medical Center, 564-1 Shimoshizu, Sakura, Chiba, 285-8741 Japan; 3 Department of Rehabilitation, Toho University Sakura Medical Center, 564-1 Shimoshizu, Sakura, Chiba, 285-8741 Japan; 4 Department of General Medical Sciences, Graduate School of Medicine, Chiba University, 1-8-1 Inohana, Chuo-ku, Chiba, 260-8677 Japan; 5 Department of Orthopaedic Surgery, Eastern Chiba Medical Center, 3-6-2 Okayamadai, Togane, Chiba, 283-8686 Japan; 6 Department of Orthopaedics, Toho University Omori Medical Center, 6-11-1 Omori-nishi, Ota-ku, Tokyo, 143-8541 Japan

**Keywords:** Reactive oxygen metabolites, Tocilizumab, Remission, Rheumatoid arthritis, Biomarker

## Abstract

**Background:**

To verify whether serum levels of reactive oxygen metabolites (ROM) are predictive of future clinical remission in patients with rheumatoid arthritis (RA) receiving tocilizumab (TCZ) therapy.

**Methods:**

A total of 46 patients with RA receiving TCZ therapy were enrolled in this study. Patients were divided into remission and non-remission groups based on disease activity score 28 (DAS28)-erythrocyte sedimentation rate (ESR) or clinical disease activity index (CDAI) at 52 weeks. Associations between serum levels of ROM, C-reactive protein (CRP), and matrix metalloproteinase-3 (MMP-3) at 4 and 12 weeks and the remission by DAS28-ESR and CDAI at 52 weeks were investigated.

**Results:**

There were no significant differences in CRP and MMP-3 between DAS- or CDAI-remission and non-remission groups at 12 weeks. However, ROM in DAS-remission group were significantly lower than those in the non-remission group. For ROM, the area under the curve of the receiver operating characteristic curve was 0.735 and the cut-off value that distinguished DAS-remission group from non-remission group was 305.5 U. Carr (sensitivity: 70.0%, specificity: 72.2%). A multivariate logistic regression analysis revealed that ROM at 12 weeks was associated with DAS-remission at 52 weeks (odds ratio: 6.067, 95% confidence interval: 1.305–28.203).

**Conclusion:**

Serum levels of ROM at 12 weeks during TCZ therapy may be predictive of DAS-remission at 52 weeks in patients with RA.

## Background

Tocilizumab (TCZ) is a humanized monoclonal antibody against the IL-6 receptor, and inhibits the binding of IL-6 to its soluble and transmembrane receptors [1, 2]. TCZ quickly reduces inflammatory reactions and significantly improves arthritis and synovitis, and prevents joint destruction in patients with rheumatoid arthritis (RA) with moderate to high disease activity that is refractory to methotrexate, several disease-modifying anti-rheumatic drugs (DMARDs) and/or tumor necrosis factor inhibitors [3]. Several large-scale clinical studies have revealed the efficacy of TCZ in patients with RA [4–7].

TCZ ameliorates inflammatory manifestations and normalizes acute phase protein levels, including C-reactive protein (CRP), thus confirming the observation that IL-6 is essential for the production of CRP [8]. Sufficient concentrations of TCZ normalize serum levels of CRP even if inflammation induced by other cytokines than IL-6 remains in joints. Therefore, clinicians sometimes encounter discrepancies between the improvements in laboratory values (e.g., negative for C-reactive protein [CRP] or normal range of erythrocyte sedimentation rate [ESR]) and the actual symptoms of patients. The problems during TCZ therapy include that clinicians have a difficulty to predict future response, and it is necessary to develop a novel biomarker other than CRP or ESR in order to predict the future clinical remission in the early treatment period.

Oxidative stress occurs in many autoimmune diseases such as RA, along with the excess production of reactive oxygen species (ROS) and reactive nitrogen species (RNS). Both ROS and RNS play a prominent role in signaling pathways in inflammatory cells and contribute to the destructive, proliferative synovitis of RA [9–13]. Recently, a d-ROM test, which measures reactive oxygen metabolites (ROM) in blood throughout the Free Radical Analytical System 4 (FRAS 4, Wismarl, Italy) [14, 15], has been developed. Using this method, we have shown that ROM serum levels were associated with CRP and disease activity score based on 28 joints (DAS28) in patients with RA [16] and that the ROM value at 12 weeks after treatment with biologic agents was a predictor of 52-week remission [17].

Regarding the efficacy of TCZ on the production of reactive oxygen species, it is reported that patients using TCZ had dramatically lower hydroperoxide levels than those using non-biological DMARDs, and that TCZ showed significantly lower ROS levels when compared to anti-TNF-α [18]. However, clinical significance of measuring ROS in patients receiving TCZ therapy has not been poorly understood. In this study, we investigated whether ROM could predict future clinical remission during TCZ therapy.

## Methods

### Patients

A total of 46 patients with RA (mean age: 60.1 y.o., disease duration: 8.1 y) receiving treatment with TCZ, including 32 naïve and 14 switched patients from other biologics, were enrolled in this study. In switched patients, a washout period of the previous biological DMARDs (bDMARDs) was not provided. Associations between the serum levels of ROM, CRP, and matrix metalloproteinase-3 (MMP-3) at 4 and 12 weeks and the remission as measured by DAS28-ESR and clinical disease activity index (CDAI) at 52 weeks were investigated.

Blood samples were collected for the measurement of serum CRP and MMP-3. In our hospital, the normal reference value for CRP is 0.3 mg/dL. ROM was also measured as described below.

To assess RA disease activity, measurements of DAS28-ESR and CDAI were obtained during the same visit at which the blood samples were collected. Remission based on the DAS28 and CDAI were defined as scores ≤2.6 and ≤ 2.8, respectively. Furthermore, a health assessment questionnaire (HAQ) was also assessed temporally.

This study was approved by the Ethics Committee of Toho University Sakura Medical Center (approval number: 2013–008), and all patients gave their written consent to participate in this study. All activities were performed in accordance with the ethical standards set forth in the Declaration of Helsinki.

### Measurement of oxidative stress markers in serum

To measure serum levels of ROM, the d-ROM test was performed using the FRAS 4 analyzer in accordance with the manufacturer’s analytical procedures [14, 15]. The measurement unit of ROM is expressed as U. Carr (Carratelli units), and the values > 300 U. Carr indicate the presence of oxidative stress.

### Statistical analyses

Results are expressed as mean ± standard deviation. All laboratory data, DAS28, CDAI, and HAQ scores were analyzed by the last-observation-carried-forward method. Between-group differences were assessed by the Mann Whitney U-test. The receiver operating characteristic (ROC) curves for ROM, CRP, and MMP-3 refer to the values at 12 weeks, and the cut-off values for ROC curves were determined by the maximum of a Youden index. A multivariate logistic regression analysis was performed by the stepwise method to compute the odds ratios (ORs) and 95% confidence intervals (95% CIs) for achievement of remission at 52 weeks. The cut-off values for ROM and CRP at 12 weeks were used as variables. All statistical analyses were performed using SPSS (ver. 19) software (SPSS, IL, USA), and *p* values < 0.05 were considered to indicate statistical significance.

## Results

### Background characteristics of patients

Background characteristics of patients included in this study are shown in Table 1. Overall, 82.6% of patients were treated with methotrexate (MTX, average 8.97 mg/week) and 58.7% with prednisolone (PSL, average 5.25 mg/day). Of the 14 patients who switched from other bDMARDs to TCZ, one had received infliximab, 2 etanercept, 5 certolizumab pegol, 4 abatacept, and 2 tofacitinib.

**Table 1:** Demographic and disease characteristics of patients

Number of patients	46
Age, years (range)	60.1 ± 13.7 (21–83)
BMI, kg/m^2^	23.1 ± 3.81
Disease duration, years (range)	8.09 ± 11.3 (1–52)
Stage I, II, III, IV	7, 17, 11, 11
RF, positive, %	71.7
ROM, U.Carr	600 ± 145
CRP, mg/dL	3.78 ± 3.77
MMP-3, ng/mL	374 ± 294
TJC	4.5 ± 4.8
SJC	3.8 ± 3.9
DAS28-ESR	4.82 ± 1.00
CDAI	17.6 ± 9.86
HAQ	0.776 ± 0.579
MTX dose, mg/week (% usage)	8.96 ± 1.53 (82.6)
PSL dose, mg/day (% usage)	5.20 ± 2.32 (58.7)

BMI, body mass index; RF, rheumatoid factor; ROM, reactive oxygen metabolites; CRP, C-reactive protein; MMP-3, matrix metalloproteinase-3; TJC, tender joint count; SJC, swollen joint count; DAS, disease activity score; ESR, erythrocyte sedimentation rate; CDAI, clinical disease activity index; MTX, methotrexate; PSL, prednisolone. Values are expressed as mean ± SD

### Changes in DAS28-ESR, CDAI, and remission rate during treatment

**Fig. 1:**
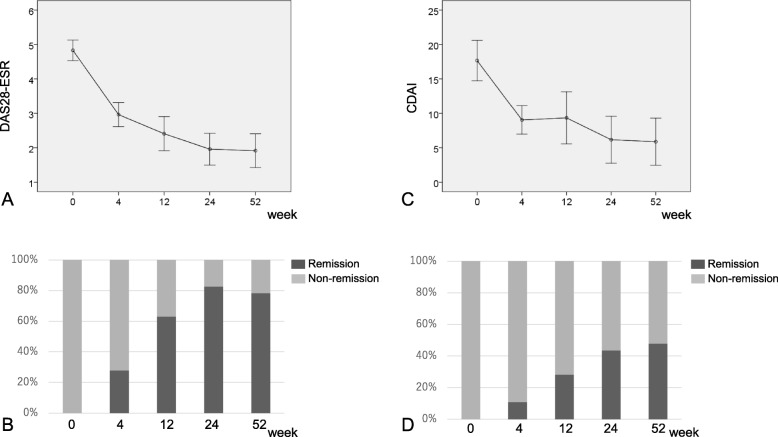
Changes in DAS28-ESR (**a**) and DAS-remission rate (**b**). Changes in CDAI (**c**) and CDAI-remission rate (**d**). DAS: disease activity score; ESR: erythrocyte sedimentation rate; CDAI: clinical disease activity index

The baseline DAS28-ESR was 4.82 ± 1.00; it rapidly decreased from baseline to 4 weeks, and further decreased gradually to 1.91 ± 1.65 at 52 weeks (Fig. 1a). The DAS-remission rate increased from baseline to 24 weeks (82.6%), and then remained the same until 52 weeks (Fig. 1b).

The baseline CDAI was 17.6 ± 9.86; it rapidly decreased from baseline to 4 weeks and further decreased gradually to 5.88 ± 11.4 at 52 weeks (Fig. 1c). The CDAI-remission rate increased from baseline to 52 weeks (47.8%) (Fig. 1d).

### Comparison of CRP, MMP-3, and ROM at 4 weeks between the remission and non-remission groups

Patients were also divided into remission and non-remission groups based on CDAI at 52 weeks. Twenty-two patients were included in the remission group and 24 in the non-remission group. There were no significant differences in CRP, MMP-3, and ROM between the DAS- or CDAI-remission and non-remission groups (Table 2).

**Table 2:** Comparison of parameters between 52 weeks-remission and 52 weeks-non remission groups at 4 weeks of TCZ therapy

	DAS			CDAI		
	R	NR	p	R	NR	p
ROM	340 ± 90.8	322 ± 65.4	0.566	343 ± 101	330 ± 67.7	0.644
CRP	0.615 ± 1.36	0.612 ± 1.00	0.995	0.454 ± 0.810	0.762 ± 1.61	0.413
MMP-3	234 ± 190	206 ± 164	0.683	204 ± 186	251 ± 182	0.411
TJC	2.7 ± 3.2	2.4 ± 2.2	0.773	1.6 ± 1.7	3.4 ± 3.4	0.052
SJC	1.8 ± 2.6	2.3 ± 2.6	0.593	1.1 ± 2.1	2.5 ± 2.8	0.094
HAQ	0.592 ± 0.532	0.825 ± 0.712	0.267	0.343 ± 0.366	0.895 ± 0.606	0.001**

TCZ, tocilizumab; ROM, reactive oxygen metabolites; CRP, C-reactive protein; MMP-3, matrix metalloproteinase-3; TJC, tender joint count; SJC, swollen joint count; HAQ, health assessment questionnaire; DAS, disease activity score; CDAI, clinical disease activity index; R, remission; NR, non-remission. Values are expressed as mean ± SD. Significantly different between remission and non-remission groups, **p < 0.01

### Comparison of CRP, MMP-3, and ROM at 12 weeks between the remission and non-remission groups

There were no significant differences in CRP and MMP-3 between the DAS- or CDAI-remission and non-remission groups. However, ROM values in the DAS-remission group were significantly lower than those in the non-remission group (*p* < 0.01, Table 3). No significant difference in ROM was seen between the CDAI-remission and the non-remission groups.

**Table 3:** Comparison of parameters between 52 weeks-remission and 52 weeks-non remission groups at 12 weeks of TCZ therapy

	DAS			CDAI		
	R	NR	p	R	NR	p
ROM	278 ± 62.4	348 ± 98.4	0.008**	276 ± 58.0	308 ± 88.4	0.158
CRP	0.150 ± 0.400	1.68 ± 0.150	0.202	0.219 ± 0.765	0.724 ± 2.28	0.329
MMP-3	120 ± 84.7	119 ± 107	0.964	105 ± 47.3	134 ± 113	0.249
TJC	2.6 ± 4.1	4.7 ± 6.6	0.23	1.7 ± 3.6	4.2 ± 5.5	0.082
SJC	1.0 ± 2.2	1.6 ± 2.7	0.505	0.41 ± 0.9	1.8 ± 3.0	0.037*
HAQ	0.500 ± 0.579	0.800 ± 0.806	0.194	0.285 ± 0.338	0.812 ± 0.739	0.004**

TCZ, tocilizumab; ROM, reactive oxygen metabolites; CRP, C-reactive protein; MMP-3, matrix metalloproteinase-3; TJC, tender joint count; SJC, swollen joint count; HAQ, health assessment questionnaire; DAS, disease activity score; CDAI, clinical disease activity index; R, remission; NR, non-remission. Values are expressed as mean ± SD. Significantly different between remission and non-remission groups, *p < 0.05, **p < 0.01

### Changes in ROM serum levels in the DAS-remission and non-remission groups at 52 weeks of treatment

Patients were divided into remission and non-remission groups based on DAS28-ESR at 52 weeks. Of the 46 patients, 36 were included in the remission group and 10 in the non-remission group.

**Fig. 2:**
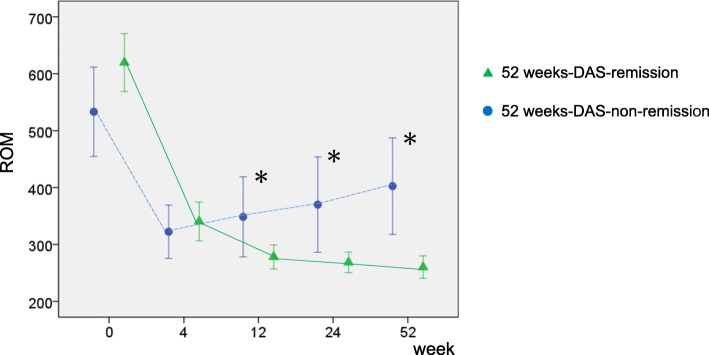
Changes in ROM serum levels in 52 weeks-DAS-remission group (straight green line) and non-remission group (dotted blue line). *Significant difference between 52 weeks-DAS-remission and non-remission group, *p* < 0.05. ROM: reactive oxygen metabolites; DAS: disease activity score

The baseline ROM serum level in the remission group was 619 ± 150 U.Carr; it decreased from baseline to 4 weeks, and then further decreased gradually to 262 ± 66.4 U. Carr at 52 weeks (Fig. 2, straight line). The baseline ROM serum level in the non-remission group was 533 ± 109 U.Carr; it decreased from baseline to 4 weeks, but then increased gradually to 402 ± 118 U. Carr at 52 weeks (Fig.2, dotted line).

### Receiver operating characteristic (ROC) analyses for ROM, CRP, and MMP-3 that distinguishes the DAS-remission group from the non-remission group

**Fig. 3:**
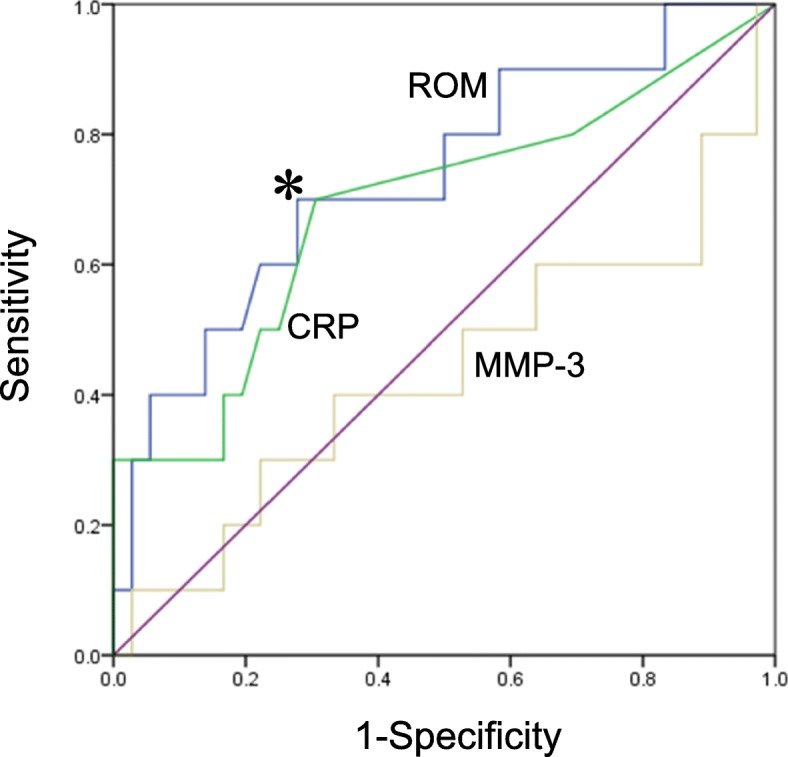
ROC curves for ROM (blue), CRP (green), and MMP-3, and cut-off value for ROM that distinguishes DAS-remission from non-remission at 52 weeks. The ROC curves refer to the values at 12 weeks. The cut-off value for ROC curves was determined by the maximum of a Youden index. An asterisk shows the cut-off value for ROM (=305.5). ROC: receiver operating characteristic; ROM: reactive oxygen metabolites; CRP: C-reactive protein; MMP-3: matrix metalloproteinase-3

**Fig. 4:**
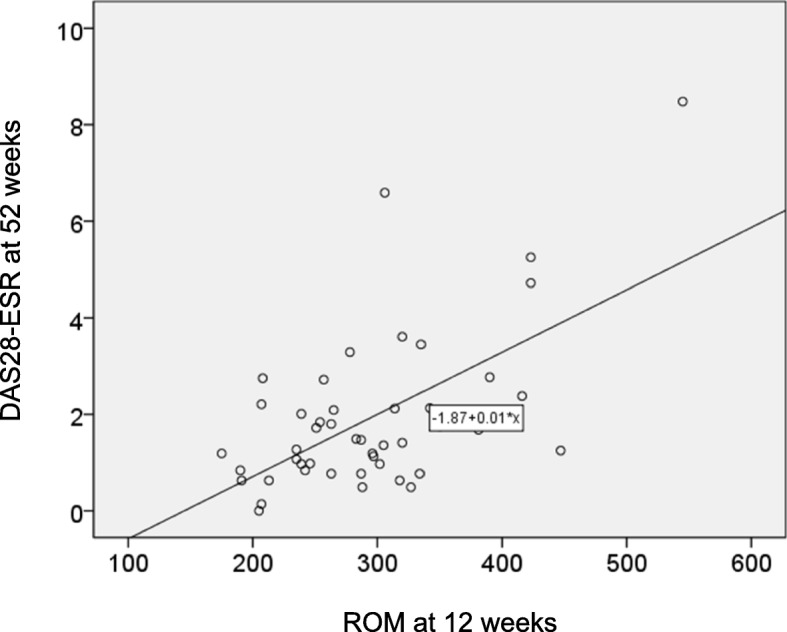
Correlation between serum levels of ROM at 12 weeks and DAS28-ESR at 52 weeks. Serum levels of ROM at 12 weeks are well correlated with DAS28-ESR at 52 weeks (*r* = 0.597, *p* < 0.01). ROM: reactive oxygen metabolites; DAS: disease activity score; ESR: erythrocyte sedimentation rate

For ROM, the area under the curve (AUC) of the ROC curve (blue) was 0.735, and the cut-off value (indicated by the asterisk) that distinguished DAS-remission from non-remission was 305.5 U. Carr (sensitivity: 70.0%, specificity: 72.2%) (Fig. 3). The AUC for CRP (green) and MMP-3 was 0.688 and 0.436, respectively. DAS-ESR at 52 weeks was highly correlated with ROM at 12 weeks (r = 0.597, *p* < 0.01) (Fig. 4).

To calculate the ORs and 95% CIs between patients with scores higher than and lower than the cut-off values, a multivariate logistic regression analysis was performed. The cut-off values for ROM and CRP at 12 weeks were used as variables. As a result, ROM at 12 weeks was identified as being associated with DAS-remission at 52 weeks (OR: 6.067, 95% CI: 1.305–28.203); however, CRP at 12 weeks was not (Table 4). Naïve or switch of bDMARDs and presence or absence of prednisolone use did not affect the association.

**Table 4:** A multivariate logistic regression analysis. The cut-off values for ROM and CRP at 12 weeks were used as variables. ORs and 95% CIs were calculated between patients with scores higher than and lower than the cut-off values. The cut-off values for receiver operating characteristic (ROC) curves were determined by the maximum of a Youden index

Variable	*p*	OR	95% CI
ROM > 305.5 vs. ≤305.5	0.021*	6.067	1.305–28.203
CRP > 0.015 vs. ≤0.015	0.155	3.306	0.637–17.153

ROM, reactive oxygen metabolites; CRP, C-reactive protein; OR, odds ratio; CI, confidence interval. *Significant differences according to a logistic regression analysis, *p* < 0.05

## Discussion

In the present study, we demonstrated that ROM serum level at 12 weeks during treatment with TCZ could be a biomarker to predict DAS-remission at 52 weeks. The cut-off value for ROM was 305.5 U. Carr, which indicates the importance of decreasing the ROM serum level to almost within normal range (< 300 U.Carr) at 12 weeks during TCZ therapy to achieve the remission.

Choosing the ideal surrogate markers for inflammation in patients receiving TCZ therapy has been controversial. ESR and CRP are well-known surrogate markers of inflammation that reflect activity of various disease states such as infection, malignancy, connective tissue disease, and coronary artery disease [19]. Particularly in RA, ESR and CRP are often used markers that reflect disease activity. However, ESR and CRP levels measured at flares in patients receiving TCZ therapy were within normal limits or lower [19]. Thus, ESR and CRP are not suitable as inflammation markers when patients are receiving TCZ therapy.

In terms of biomarkers to evaluate the clinical course during treatment with TCZ, it has been reported that serum IL-6 is a useful marker instead of CRP and ESR because TCZ is an antibody to the IL-6 receptor that does not directly suppress IL-6 production [3]. The researchers showed that serum IL-6 levels from 12 to 24 weeks after TCZ initiation better reflected the efficacy of TCZ at 52 weeks. It has also been reported that the neutrophil-to-lymphocyte ratio is a more reliable marker than ESR or CRP for evaluating the disease activity in patients with RA during TCZ therapy [19].

Recently, several studies have documented predictors of response to TCZ at baseline. Discontinuation of TCZ within 1 year was predicted by low CRP, high HAQ and prior exposure to biological agents [20], while younger age, baseline high CRP levels and no history of prior cardiovascular events were predictors of better response to TCZ [21]. However, predictors in the early stage after starting TCZ therapy have not been demonstrated to date.

Serum IL-6 level before and after TCZ therapy is a principal biomarker in patients with RA [22]. Baseline serum IL-6 level is a potential biomarker reflecting disease activity. Furthermore, serum IL-6 level during TCZ therapy is a useful biomarker to estimate residual inflammation in joints and to predict responsiveness to TCZ. Although measurement of serum IL-6 appears to be useful for judging the treatment process and predicting future remission, it is performed by enzyme immunoassay or enzyme-linked immunosorbent assay that generally need high costs and several days to receive the results.

Measurement of ROM is easily performed using FRAS 4 analyzer with low costs, and investigators can know the results shortly. Instead of IL-6, serum ROM level was measured in this study; at 12 weeks, it was significantly lower in the DAS-remission group than in the non-remission group. At that time, CRP and MMP-3, two conventionally used biomarkers for monitoring the disease activity of RA, were also compared between the DAS-remission and non-remission groups, but there were no significant differences. A multivariate logistic regression analysis revealed that ROM at 12 weeks was associated with DAS-remission at 52 weeks, but CRP at 12 weeks was not. These observations suggest that ROM at 12 weeks is predictive of DAS-remission at 52 weeks during treatment with TCZ, and that ROM is superior to CRP or MMP-3 as a surrogate marker to predict DAS-remission.

In patients receiving TCZ therapy, CDAI is often used to evaluate disease activity. However, there were no significant differences in CRP, MMP-3, and ROM at 12 weeks between the CDAI-remission and non-remission groups. Why was the serum level of ROM not predictive of CDAI-remission? We previously showed that the serum level of ROM correlated with CRP in RA patients [16]. Since CDAI does not contain an index of inflammation such as CRP or ESR, CDAI may not be associated with ROM. Actually, in terms of SDAI- or Boolean-remission, both of which contain CRP, there was a significant difference in ROM levels at 12 weeks between the remission and non-remission groups (data not shown). Another possibility is the small number of patients. Since there was only a tendency that the ROM level at 12 weeks of 52 weeks-CDAI-remission group was lower than that of 52 weeks-CDAI-non remission group (*p* = 0.158, Table 3), further studies are required to conclude if the serum level of ROM could be predictive of 52 weeks-CDAI-remission.

We previously reported that the cut-off value of ROM at 12 weeks that distinguished DAS-remission from non-remission in patients receiving biological DMARDs (bDMARDs) was 381.5 U. Carr [17], although it was 305.5 U. Carr in patients receiving TCZ in this study. Since TCZ strongly suppresses production of CRP, and ROM is well correlated with CRP, the cut-off value of ROM would be lower than when using bDMARDs other than TCZ. Since the normal range of ROM is < 300 U. Carr, it is important to decrease ROM levels to within the normal limit at 12 weeks to achieve remission at 52 weeks.

This study has some limitations. First, the sample size was small. An investigation using a large sample size will be necessary to establish whether ROM is predictive of remission. Second, although oxidative stress is involved in the pathophysiology of many diseases in humans [23–28], its role in the pathobiology of RA remains unclear. Further studies are required to establish the clinical significance of oxidative stress in RA.

## Conclusion

We demonstrated that the ROM serum level at 12 weeks during treatment with TCZ was a useful biomarker to predict DAS-remission at 52 weeks. As a biomarker to predict DAS-remission, ROM was superior to CRP or MMP-3. The serum level of ROM could be a biomarker to monitor treatment efficacy of TCZ in routine clinical practice.

## Data Availability

All data generated or analyzed during the current study are included in this published article.

## Abbreviations

CDAI: clinical disease activity index
CRP: C-reactive protein
DAS 28: disease activity score based on the examination of 28 joints
HAQ: health assessment questionnaire
MMP-3: matrix metalloproteinase-3
RA: rheumatoid arthritis
ROM: reactive oxygen metabolites
TCZ: tocilizumab
